# TransSiamUNet based transformer-augmented Siamese-U-Net for precise change detection in satellite imagery

**DOI:** 10.1038/s41598-026-43164-w

**Published:** 2026-04-07

**Authors:** Farid Ali, Soha Safwat Labib, Ayat Mahmoud, Ibrahim Eldesouky Fattoh

**Affiliations:** 1 Information Technology Department, Faculty of Computers and Artificial Intelligence, Beni-Suef University, Beni Suef, Egypt; 2 Software Engineering and Information Technology, The Egyptian Chinese University, Cairo, Egypt; 3 Computer Science, October University for Modern Sciences and Arts, Giza, Egypt; 4 Computer Science Department, Faculty of Computers and Artificial Intelligence, Beni-Suef University, Beni Suef, Egypt

**Keywords:** Change detection, Satellite imagery, Urban development, Siamese network, Vision Transformer (ViT), Engineering, Environmental sciences, Mathematics and computing

## Abstract

Identifying changes in satellite images is vital for tasks like tracking land cover and land use, evaluating disaster impacts, and conducting military surveillance. Although conventional techniques for detecting changes in multispectral remote sensing data are commonly applied, they often fail to meet the requirements for reliability and precision. Recently, deep learning methods have emerged, providing more accurate and effective solutions for monitoring environmental transformations and urban expansion in satellite imagery. This paper introduces TransSiamUNet, a deep learning architecture that combines Siamese networks, U-Net segmentation, and Vision Transformers (ViT) for high-precision change detection. The model processes paired Sentinel-2 images via a tailored preprocessing pipeline and integrates local and global feature extraction for pixel-level change segmentation. On the OSCD benchmark, TransSiamUNet achieves an accuracy of 0.94, surpassing the Siamese network (0.86), U-Net (0.84), and Siamese+U-Net hybrid (0.91). These results demonstrate the model’s superior capability in detecting fine-grained urban and environmental changes, highlighting its suitability for real-world remote sensing applications.

## Introduction

Change detection in satellite imagery is a fundamental task in remote sensing, supporting critical applications such as urban planning, disaster assessment, and environmental monitoring. Traditional pixel-based methods–including image differencing, ratioing, and regression–rely on spectral or intensity comparisons^1,2^. While computationally efficient, these approaches are sensitive to noise, illumination variations, and misalignment, limiting their reliability in complex real-world scenarios.

Deep learning has revolutionized the field by enabling automatic feature learning. Siamese networks^3^ and fully convolutional architectures like U-Net^4^ have achieved improved accuracy by extracting spatial-semantic features. Recent advances further incorporate attention mechanisms^5^ and transformer-based models^6,7^ to capture long-range dependencies. More recently, State Space Models (SSMs) such as Mamba^8^ have emerged as efficient alternatives for sequence modeling in remote sensing tasks.

Change detection technologies are widely adopted for monitoring urban environments, enabling the identification of new construction, infrastructure upgrades, and the transformation or loss of green spaces. These methods support urban planners, developers, and local authorities by providing timely insights into the evolving landscape of cities^9^.

The increasing frequency of climate change-driven disasters such as heat waves, storms, floods, and droughts has highlighted an urgent research challenge and the necessity for more advanced, automated change detection techniques. Traditional methods often struggle with real-time accuracy and require manual input, while recent deep learning approaches, especially those leveraging Synthetic Aperture Radar (SAR) data combined with novel architectures like enhanced U-Net models incorporating attention mechanisms, show promise in improving detection precision and adaptability across diverse environments^10^.

Deep learning approaches have been adopted for change detection in remote sensing, showing strong performance and enabling the automatic identification of changes in satellite imagery^11^.

Deep learning techniques have recently been applied to remote sensing, achieving impressive results across various tasks. Several review articles have summarized advancements in this area, highlighting deep learning’s effectiveness in image classification, object detection, denoising, scene interpretation, and other key applications within remote sensing^12^.

Processing satellite images presents distinct challenges, primarily because their characteristics differ significantly from those of conventional images. Factors such as large data size, acquisition from great distances, and increased noise levels make analysis and interpretation more complex than with traditional imagery^13^.

One significant limitation in satellite image analysis is the relatively small size of detectable objects due to coarse spatial resolution. For example, while a football stadium might occupy hundreds of pixels in a ground-level photograph–allowing for detailed analysis–the same stadium could be represented by only a handful of pixels in a satellite image with a 10-meter resolution. This reduction in spatial detail hinders accurate object detection and classification^14^.

Satellite images are often affected by environmental conditions present during capture. Factors such as clouds, haze, and shadows can hide important features in the imagery, making advanced preprocessing essential to reduce these impacts and retrieve useful information^15^. Differences in the position of the sun when satellite images are taken can cause variations in lighting and shadowing, especially around tall buildings and mountainous areas. These changes in illumination can be mistaken for actual alterations in the landscape when using basic image processing approaches, making it more challenging to accurately identify genuine changes over time^16^.

While standard images are typically limited to three color channels–red, green, and blue–satellite hyperspectral images can capture information across hundreds of spectral bands. This extensive spectral resolution offers valuable data for many uses, but it also makes data processing and analysis much more complex, demanding the use of advanced techniques for feature extraction and dimensionality reduction^17^.

The accuracy and usefulness of satellite images can be significantly reduced by atmospheric conditions such as clouds, haze, and fog, which may hide important surface details. To overcome these challenges and ensure reliable analysis, it is crucial to apply advanced preprocessing techniques like cloud detection, removal, and atmospheric correction to improve image quality and reveal relevant information^18^.

The challenges mentioned above emphasize the importance of employing advanced and dependable processing methods and algorithms for satellite imagery, which are essential for effective environmental monitoring, land-use mapping, and managing disaster response efforts. Change detection is a core technique in earth observation, designed to distinguish between changed and unchanged pixels in remote sensing images captured at different times from the same area. The main goal is to assign each pixel a binary label “unchanged” or “changed” based on pairs or sequences of co-registered images. This approach is crucial for multi-temporal analysis, urban mapping, and monitoring dynamic environments^19^.

Change detection techniques are widely applied across various fields. In agriculture, they are used to monitor crops, assess the impact of disasters, and track deforestation. The military sector benefits from change detection for observing troop activities, identifying new structures, and evaluating damages. Urban planners use these methods to oversee city development and changes in land use. Additionally, environmental researchers utilize change detection to investigate the consequences of climate change, such as glacier shrinkage, rising sea levels, and alterations in vegetation^20^.

**Recent advances:** Beyond Transformers, State Space Models (SSMs) such as Mamba have emerged as a powerful alternative for sequence modeling, offering linear complexity and strong long-range dependency capture. In remote sensing,^21^ introduced Algae-Mamba for algal bloom extraction, demonstrating its spatial adaptability. More directly relevant,^8^ proposed ChangeMamba, a spatiotemporal SSM for remote sensing change detection, achieving competitive accuracy with reduced computational overhead. While Mamba-based methods show promise, hybrid architectures that combine convolutional symmetry, global attention, and multi-scale decoding–as proposed in TransSiamUNet–remain under-explored and offer complementary strengths for precise change mapping. Despite these advancements, existing methods face three key limitations: (1) Siamese CNNs lack global context; (2) pure transformers lose fine spatial details due to coarse tokenization; and (3) hybrid designs often treat modules independently without synergistic fusion. To address these gaps, we propose **TransSiamUNet**, a unified architecture that integrates a weight-shared Siamese encoder for symmetric feature extraction, a Vision Transformer (ViT) for global dependency modeling in the difference domain, and a U-Net decoder with cross-scale skip connections for precise spatial localization. This design ensures that local discriminability, global context, and boundary accuracy are jointly optimized.

The main contributions of this work are:

- A novel hybrid architecture that effectively combines Siamese feature learning, transformer-based global attention, and U-Net segmentation in a coherent framework.
- Rigorous ablation studies quantifying the contribution of each component and their synergistic interactions.
- Extensive experiments on multiple benchmarks (OSCD, LEVIR-CD, WHU) demonstrating state-of-the-art performance and strong generalization.
- Detailed implementation specifications to ensure reproducibility, including architecture details, training protocols, and post-processing configurations.

## Traditional techniques for change detection in satellite imagery

Basic change detection techniques in remote sensing work by comparing two satellite images taken at different times to identify differences. These methods analyze spatially aligned images to detect variations in features such as shape, size, position, and identity, enabling the tracking of changes over time in various applications.

### Image differencing

Image differencing is a technique in image processing where two images taken at different times are compared by subtracting the pixel values of one image from the other. This process generates a new image that highlights the differences between the two, making it possible to identify areas where significant changes have occurred. The method is straightforward and effective for quickly detecting changes, but its accuracy depends on precise alignment of the images and can be affected by unrelated variations such as noise or atmospheric effects^22^.

1 $$\begin{aligned} I_{\text {diff}} = \left| \textrm{Img}_i^2 - \textrm{Img}_i^1 \right| \end{aligned}$$

As shown in Equation 1, image differencing between two images is a method used to identify changes between two images by calculating the absolute differences in their pixel values at corresponding points. This approach is often employed to detect variations that have occurred over time. Although it is a fast and simple technique for highlighting changes, it can be affected by noise and may have difficulty distinguishing meaningful changes from minor variations caused by environmental conditions or sensor inconsistencies^22^.

### Image ratioing

Image ratioing is a commonly used method in remote sensing for detecting changes. It works by dividing the pixel values of one image by those of another, comparing them band by band. Significant changes are revealed when the ratio values differ greatly from one.

2 $$\begin{aligned} \text {Rationing} = \left| \frac{\textrm{Img}_i^2}{\textrm{Img}_i^1} \right| \end{aligned}$$

This approach Equation 2 is valued for being quick and easy to apply. However, the resulting ratio images as shown in equation 2 often have histograms that are not normally distributed, which can make it harder to spot minor changes and interpret results, especially in areas with subtle differences or low contrast^23^.

### Normalized difference built-up index

The Normalized Difference Built-up Index (NDBI) is commonly applied to track urban development by comparing the spectral characteristics of the NIR and SWIR bands as shown in equation 3. By computing the NDBI for images taken at different times and analyzing the changes between them, it becomes possible to identify areas where cities have expanded.

3 $$\begin{aligned} \text {NDBI} = \frac{\textrm{SWIR} - \textrm{NIR}}{\textrm{SWIR} + \textrm{NIR}} \end{aligned}$$

This method displayed in Equation 3 is particularly effective at highlighting built-up regions because it reduces the impact of terrain and lighting differences. However, it can also increase the presence of noise or unrelated signals, which may cause errors or false detections, especially outside of urban zones^24^.

### Average intensity

AI-based normalization methods for change detection frequently work by modifying the average brightness of pixels across images taken at different times. This makes them particularly effective at detecting changes in areas with limited light, such as shadows or thick vegetation.

4 $$\begin{aligned} \text {AI} = \frac{\textrm{Img}_i^2 - \textrm{Img}_i^1}{\textrm{Img}_i^2 + \textrm{Img}_i^1} \end{aligned}$$

Equation  4 show the average intensity; Their main advantage is the ability to pick up on minor differences in dimly lit regions. However, when environmental conditions–like atmospheric changes or sensor inconsistencies–vary greatly, these methods often need additional statistical or adaptive components to ensure accurate results, which can make the overall process more complex^25^.

### Euclidean distance

The Euclidean distance method for detecting changes works by measuring the direct distance between pixel values in the spectral space of images taken at different times as shown in equation 5. Larger distances typically signal more significant changes, while smaller ones suggest little or no change.

5 $$\begin{aligned} \text {ED} = \sqrt{ \sum _{i=1}^n \left( \textrm{Img}_i^2 - \textrm{Img}_i^1 \right) ^2 } \end{aligned}$$

This technique in Equation  5 is popular because it is easy to understand and apply. However, it may have difficulty identifying minor changes or when the spectral properties of different land cover types are similar, which can lead to unclear or unreliable results^26^.

### Image regression

A regression model is used to uncover and quantify the relationship between a dependent variable and one or more independent variables. By fitting the model to paired data, it can predict values for the dependent variable based on the independent variables. The differences between these predicted values and the actual observed values provide insights into the quality of the model fit and can be used to detect patterns, anomalies, or changes within the data. Image regression works by modeling the relationship between two images through a regression function applied to the pixel values of corresponding locations in each image. This function is used to predict the pixel values of the second image from the first, typically in the form

6 $$\begin{aligned} \text {IR} = aI + b \end{aligned}$$

In Equation  6 , I is the pixel value from the first image, a is the slope, and b is the intercept. By comparing the predicted values to the actual pixel values in the second image, differences can be identified and used for change detection. Regression-based techniques are effective at minimizing differences caused by variations in sensors and radiometric properties between images, which enhances the reliability of change detection in diverse scenarios. However, a significant challenge of these methods is the necessity to carefully design and calibrate regression models for each spectral band. This process can be particularly difficult and time-consuming when working with images from different sensors or under changing environmental conditions^27^.

Conventional change detection methods are valued for their simplicity and speed, but they are often hindered by issues such as noise, inconsistencies in sensor data, and environmental changes. These shortcomings have encouraged the adoption of more sophisticated approaches, like machine learning and deep learning, which can automatically extract complex features and provide greater resilience and accuracy across a variety of conditions^28^.

## Comprehensive literature review on change detection in remote

This survey provides an overview of 25 sample works in the field of change detection (CD) in remote sensing photos. These works are divided into three primary categories: (1) Traditional Machine Learning–Based Methods, (2) Deep Learning Models, and (3) Transformer-Based Methods. Every document is broken down into a paragraph that includes information on the fundamental model or method, the datasets that were utilized, the primary findings that were published (accuracy, F1, and Kappa, if reported), the most important advantages, and the most significant shortcomings.

### Traditional machine learning–based methods

Wessels et al. (2016) introduced a hybrid pipeline that integrates Change Vector Analysis (CVA) with a Random Forest (RF) classifier for the detection of land-cover change in multispectral data. The method initially calculates per-pixel change vectors across dates, minimizes spectral redundancy using PCA, then inputs the selected difference features into a Random Forest that generates change/no-change labels. The method assessed using Landsat-8 imagery in agricultural areas had an overall accuracy of approximately 85%, surpassing basic CVA and simple thresholding benchmarks. Advantages: straightforward, comprehensible, few data prerequisites, rapid training and inference for medium-scale environments. Weaknesses: susceptible to spectral noise, inadequate at simulating intricate patterns (mixed pixels) or slight structural alterations^29^.

Nielsen et al. (2022) presented an unsupervised framework combining PCA and KMeans for change detection in high-resolution optical images. PCA compresses spectral data and emphasizes principal components, while KMeans categorizes pixels into change and no-change groups utilizing difference-space features. The technique attained almost 80% accuracy on QuickBird urban datasets, establishing a robust unsupervised baseline. Advantages: necessitates no labeled data (beneficial for novel domains), straightforward to execute. Weaknesses: susceptibility of KMeans to initialization, low resilience to seasonal variations and changes in light^30^ .

Li et al. (2017) devised a spectral-textural random forest and fuzzy set-based detector that integrates hand-crafted features (spectral indices, GLCM texture) and differential measurements to distinguish change vs no-change. Assessed using SPOT-5 imagery, the results indicate approximately 82% overall accuracy, showcasing commendable precision in scenarios with limited labeled data. Strengths: operates effectively with constrained labeled data and interpretable attributes. Limitations: necessitates meticulous feature engineering and exhibits suboptimal scalability with several sensors/modalities^31^.

Zhou et al. (2020) utilized Random Forests for monitoring forest changes through VDVI/time-series derived features. Their pipeline employed temporal differencing of vegetation indicators in conjunction with RF classification on MODIS and Sentinel-derived NDVI series, achieving overall accuracies of approximately 84% for deforestation and afforestation identification in the examined regions. Advantages: comprehensible, efficient for vegetative dynamics. Weaknesses: difficulties with varied land cover compositions and minor disruptions^32^.^33^ introduced a graph-based unsupervised change detection methodology in which pixels serve as nodes, and spectral/spatial similarity establishes edges; a graph partitioning/cut technique discerns consistent changing clusters across time. The approach applied to Sentinel-2 urban scenes yielded a Kappa of approximately 0.81, outperforming less effective clustering baselines. Strengths: explicitly encodes spatial structure and neighborhood coherence; beneficial when training labels are absent. Weaknesses: computational cost for extensive images (graph size), vulnerability to hyperparameters in graph creation Ekaterina et al. (2020).

### Deep learning models

Zhu et al. (2018) introduced an initial Siamese-CNN architecture for bi-temporal change detection, wherein two image patches (from date A and date B) are processed by a shared CNN encoder. The extracted features are then compared (via difference or concatenation) and forwarded to a classifier head that generates per-pixel change predictions. Assessed using QuickBird high-resolution pairs, they reported approximately 88% overall accuracy, demonstrating the superiority of learnt feature representations compared to manually generated ones. Strengths: acquires resilient joint representations, minimizes feature engineering. Vulnerabilities: susceptibility to misregistration and significant scale changes; requires labeled pairings^3^.

Daudt et al. (2018) presented three fully convolutional Siamese models (FC-EF, FC-Siam-concat, FC-Siam-diff) that function end-to-end on complete pictures utilizing encoder–decoder U-Net type architectures and several fusion methodologies (early fusion, Siamese concatenation, and feature difference). They assessed performance on the OSCD benchmark and several high-resolution datasets, indicating enhancements in F1 of approximately +10 percentage points compared to traditional patch-based baselines, and facilitating near real-time inference (reported at <0.1 s per tile in certain configurations). Strengths: comprehensive training, streamlined architecture, rapid inference, significant baseline. Weaknesses: restricted long-range context in superficial versions, predominantly binary output (change/no-change) lacking semantic descriptors^4^.

Mou et al. (2017) advocated for the application of recurrent neural networks (LSTM/GRU) to model spectral sequences in hyperspectral change detection, wherein spectral bands or reduced spectral vectors are seen as temporal sequences, allowing RNNs to learn the spectral transitions linked to change. The method applied to AVIRIS hyperspectral temporal pairings achieved an overall accuracy of approximately 84% and enhanced sensitivity to minor spectral variations. Advantages: explicitly models spectrum relationships, effective for hyperspectral imaging modalities. Weaknesses: The training of RNNs is slower, and the models exhibit reduced scalability at extremely high spatial resolutions^4^ .

Chen et al. (2020) – STANet introduced a Spatio-Temporal Attention Network that enhances a Siamese encoder with attention modules designed to align and highlight corresponding regions over time. Upon evaluation using the LEVIR-CD building change dataset, STANet achieved an F1 score of around 83.5%, demonstrating reduced false positives and superior recognition of small objects compared to standard U-Net baselines. Strengths: enhanced attentiveness facilitates correspondence and sensitivity to little objects. Disadvantages: increased computing expense and greater training complexity^5^.

Gong et al. (2017) examined conditional GANs for change detection, wherein the generator produces change maps (or translates from date A to date B), while a discriminator ensures realism and consistency. The GAN-based algorithms applied to Landsat multispectral scenes achieved an overall accuracy of approximately 86%, producing dramatically enhanced change maps. Strengths: capable of modeling intricate change distributions and generating realistic masks; advantageous for unsupervised or weakly supervised contexts. Weaknesses: Instability in GAN training (mode collapse), risk of hallucinations^34^.

Wu et al. (2020) utilized Variational Autoencoders (VAE) for SAR change detection, training models to reconstruct patches from one date, with reconstruction residuals from encoding the other date signifying changes; the method achieved a Kappa of approximately 0.78 on Sentinel-1 test sites and demonstrated enhanced robustness to speckle. Strengths: autonomous (or semi-autonomous) functionality, aids in mitigating SAR noise. Weaknesses: the quality of reconstruction constrains sensitivity to nuanced variations; interpretation of latent space is less robust^35^.

Fang et al. (2021) – SNUNet-CD introduced a Siamese Nested U-Net architecture including dense nested skip connections and robust multi-scale fusion, designed explicitly for building change detection. In LEVIR-CD, they reported an F1 score of approximately 90.5%, indicating significant enhancements in boundary sharpness and the memory of small objects. Strengths: exceptional preservation of multi-scale detail, robust boundary performance. Weaknesses: extensive parameter count, increased GPU memory consumption, and susceptibility to overfitting in the absence of augmentation^36^.

Ding et al. (2022) enhance semantic change detection (SCD) by pinpointing both the areas of change and the corresponding land-cover/land-use classifications. Previous CNN models featuring triple-branch topologies exhibited restricted interaction among the branches. To address this issue, Bi-SRNet was introduced, incorporating semantic reasoning blocks and an innovative loss function, resulting in enhanced accuracy and consistency in the detection and segmentation of changes. When assessed on the SEmantic Change detectiON Dataset (SECOND), Bi-SRNet achieved an overall accuracy of around 87.84%. Strengths: demonstrates proficiency in identifying essential areas. Weaknesses: heightened model complexity and a multi-phase training pipeline^37^.

The multi-scale attention Siamese CNN (MASNet) was put forth by Li and colleagues (2022) , which integrates channel and spatial attention in parallel branches and fuses multi-scale data through a pyramid. The model reported an overall accuracy of approximately 91.3 percent on WHU-CD and other datasets with a high resolution of building alteration, which enhanced the robustness of the scale fluctuation. Advantages: is capable of dealing with construction site footprints that vary in scale and clutter. Deficiencies: It is necessary to perform hyperparameter adjustment on attention modules, and they also increase computation^38^.

Shu et al. (2022) – DPCC-Net presented a Dual-Perspective Change Contextual CNN that preserves complementary local and global contextual streams and merges them using context aggregation blocks. The approach was able to get an F1 score of approximately 92 percent on LEVIR-CD+, demonstrating robust generalization across scenes. Benefits: more in-depth contextual modeling, excellent performance in chaotic urban environments Shortcomings: a significant amount of memory is required, and training takes a longer period of time^39^.

Zhang et al. (2022) developed a dual-attention ResNet variation that used spatial and channel attention blocks to improve Siamese characteristics prior to differencing. The system reported an overall accuracy of approximately 90.8% on WHU-CD and a reduction in false alarms in darkened regions. Strong points: an appropriate balance between contextual and local factors Disadvantages: when compared to lightweight CNNs, it has a longer inference time^40^.

Liu et al. (2022) proposed a context-aggregation convolutional neural network (CNN) that incorporates multi-scale context using atrous/dilated convolutions and feature fusion. They achieved an F1 score of about 91.2% on the LEVIR-CD+ dataset, as well as consistent performance under moderate misregistration. Strong points: the ability to withstand registration errors and local noise Disadvantages: a longer training period and more extensive parameter settings^41^.

Sun et al. (2023) introduced a lightweight CNN architecture designed for UAV/near-real-time change detection, utilizing depthwise separable convolutions and efficient decoder heads; the model attained approximately 88% overall accuracy on UAV datasets while facilitating real-time performance on embedded GPUs. Strengths: efficacy and relevance for edge devices. Weaknesses: compromise between absolute precision and cumbersome models^42^.

### Transformer-based methods

Bandara and Patel (2022) – ChangeFormer unveiled a Siamese transformer encoder with cross-attention blocks that facilitate bi-temporal interactions at the token level. The model segments picture patches, employs shared transformer encoders, and utilizes cross-attention to directly compare tokens across dates; a decoding head generates per-pixel change masks. ChangeFormer, assessed on LEVIR-CD and additional benchmarks, achieved an F1 score of approximately 91%, surpassing numerous CNN baselines because to its long-range context modeling capabilities. Strengths: encapsulates global interdependencies and intricate contextual relationships. Weaknesses: increased memory and computational requirements, necessitates larger training datasets or pretraining^6^.

A multi-scale Swin Transformer backbone with deep supervision (multi-stage decoding) was suggested by Song et al. (2022) as a part of the MSTDSNet-CD (Swin based) approach in order to generate precise change masks. Transformer layers are able to capture context, and the sliding-window self-attention in Swin gives localization. On the LEVIR-CD+ dataset, MSTDSNet-CD reported an F1 score of approximately 92.5 percent. Positive aspects: effective multi-scale modeling and convergence that are both exceptional. Shortcomings: the window overhead and the high memory requirements of extremely large images^43^

Li et al. (2023) designed a hierarchical transformer with multi-level fusion that utilizes coarse tokens to offer global guidance and fine tokens to maintain spatial detail. Experiments conducted on WHU-CD showed that the overall accuracy (OA) was approximately 92 percent, with improved false positive control in textured areas. Strong points: the merging of features with several levels of granularity; Deficiencies: a complicated architecture and training process^44^.

Jiang et al. (2023) introduced VcT (Visual change Transformer), which included token mining (top-K reliable tokens), graph token generation, and anchor-primary attention in order to improve the localization of changes. These techniques were evaluated on a number of benchmarks that examined changes in buildings (for example, LEVIR-CD variations). In terms of the suppression of unaltered background, VcT reported a score of approximately 91–92 percent, which was exceptionally impressive. Benefits: the process of mining tokens in an organized manner that takes advantage of places that have not been modified in order to maintain stability Disadvantages: Performing operations on graphs increases the amount of overhead and the level of complexity involved in the implementation^45^.

ChangeViT, as described by Cao et al. (2024) , is a technique that use a plain Vision Transformer (ViT) as the foundation for the purpose of change detection. However, it also incorporates a detail-injector module that reintroduces high-frequency spatial detail back into patch tokens, thereby offsetting the coarse tokenization that is characteristic of ViT. On the LEVIR-CD and WHU-CD datasets, ChangeViT achieved an overall accuracy of approximately 93.1 percent and an F1 score of approximately 92.7 percent, which placed it in the state-of-the-art category or the near-state-of-the-art category on these sets. Advantages: a straightforward foundation (plain ViT) that is combined with a module that is successful at bringing back fine details; Shortcomings: ViT-style models necessitate large-scale pretraining or extensive training datasets, and they also entail a high computational expense^46^.

A two-stage transformer architecture was proposed by Zhong et al. (2025) LRNet (Localization-Then-Refinement)–that included (1) a localization encoder that predicted coarse change regions using multi-branch encoders (including differencing branches and a Learnable Optimal Pooling operator), followed by (2) an edge-aware refinement decoder that aligned and sharpened boundaries (Edge-Area Alignment). The results of LRNet on Sentinel-2 urban datasets were an overall accuracy of around 94.2% and an F1 score of approximately 93.8%, with notable benefits in terms of boundary correctness and tiny object delineation. Benefits: the ability to achieve good precision in boundaries and robust localization through the use of multi-stage processing Shortcomings: increased inference time and architectural complexity as a result of the two steps^47^.

Contrastive Dual-Stream Swin Transformer by Wang and colleagues (2025) The dual-stream architecture that was established by pretraining consists of two Swin backbones for dates A and B, respectively. This architecture fuses cross-temporal tokens and benefits from a contrastive pretraining stage that helps stable representations across dates and sensors. They reported an F1 of approximately 94.1 percent on LEVIR-CD+ and mixed urban datasets, demonstrating that they had achieved better robustness in terms of both sensor noise and seasonal change. Strong points: the combination of contrastive pretraining and dual-stream cross attention results in resilience. Shortcomings: its need on extensive pretraining corpora and the prohibitively high expenses associated with training and inference^48^.

Recent transformer-based architectures have shown strong performance in remote sensing change detection. For instance,^49^ introduced a pure transformer model for change detection, demonstrating superior capability in modeling long-range dependencies.^50^ proposed TransUNet, a hybrid architecture combining transformers with U-Net for medical image segmentation, inspiring similar designs in remote sensing for improved feature representation.

Handling complex remote sensing scenes–such as those with cloud occlusion, seasonal variations, and misalignment–remains a critical challenge.^51^ proposed a gated local-global fusion approach (GLGF-CR) for cloud removal, enhancing feature robustness in obscured regions.^43^ developed SwinSUNet, a pure transformer network leveraging Swin Transformer for change detection, achieving state-of-the-art results through hierarchical feature learning. Additionally,^52^ explored style transformation-based spatial–spectral feature learning for unsupervised change detection, effectively addressing domain shifts and seasonal variations.

These advances underscore the importance of global context modeling, multi-scale fusion, and robust feature alignment–key aspects that TransSiamUNet integrates through its Siamese-ViT-U-Net design, positioning it as a holistic solution for precise and reliable change detection in varied remote sensing conditions.

Table 1, which describes the evolution from conventional techniques to deep learning and transformer-based models, summarizes the major advancements in change detection. While deep learning algorithms greatly increase performance by learning spatial-spectral representations, they come at a larger computational cost. Traditional techniques, on the other hand, exhibit limited accuracy and mainly rely on handcrafted features. Although they still require a lot of resources, recent transformer architectures further improve accuracy through multi-scale fusion and long-range attention. Overall, the table shows a distinct trend toward models that are more powerful but require more processing power.

**Table 1:** Summary of traditional techniques for change detection in satellite imagery.

Study – Model - Year	Dataset(s)	Reported Metric	Strengths	Limitations
VA + RF (Wessels) - Traditional ML, 2016^29^	Landsat-8	OA = 85%	Simple, interpretable	Noise sensitivity
PCA + KMeans (Chen), Traditional ML, 2022^30^	QuickBird	Acc = 80%	Unsupervised	Init sensitivity
RF and Fuzzy set + features (Li), Traditional ML, 2017^31^	SPOT-5	OA =82%	Works with few labels	Feature engineering
RF + NDVI (Zhou), Traditional ML, 2021^32^	MODIS-Sentinel	OA =84%	Vegetation focus	Heterogeneity issues
Graph-based (Ekaterina), Traditional ML, 2020^33^	Sentinel-2	Kappa =0.81	Spatial modeling	Expensive
Siamese CNN (Zhu), Deep Learning, 2015^3^	QuickBird	OA =88%	Learned features	Alignment sensitivity
FC-Siam (Daudt), Deep Learning, 2018^4^	OSCD	F1 +10% vs baselines	End-to-end, fast	Local context only
RNN HSI (Mou), Deep Learning, 2017^4^	AVIRIS HSI	OA =84%	Spectral modeling	Slow training
STANet (Chen), Deep Learning, 2020^5^	LEVIR-CD	F1 =87.3%	Temporal attention	Costly
GAN CD (Gong), Deep Learning, 2017^34^	Landsat	OA =86%	Realistic maps	GAN instability
VAE SAR (Wu), Deep Learning, 2020^35^	Sentinel-1	Kappa =0.78	Speckle suppression	Interpretability
SNUNet-CD (Fang), Deep Learning, 2021^36^	LEVIR-CD	F1 =90.5%	Fine detail	Heavy model
Bi-SRNet (Zheng), Deep Learning, 2022^31^	SECOND	OA =87.84%	Discriminating critical areas	Complex pipeline
MASNet (Li), Deep Learning, 2022^38^	WHU-CD	OA =91.3%	Multi-scale	Tuning sensitive
DPCC-Net (Shu), Deep Learning, 2022^39^	LEVIR-CD+	F1 =92%	Contextual cues	Memory heavy
Dual-attention ResNet, Deep Learning, 2022^40^	WHU-CD	F1 =90.8%	Local/global balance	Slow
Context aggregation, Deep Learning, 2022^41^	LEVIR-CD+	F1 =71.29%	Robustness	Heavy params
Lightweight UAV CNN (Sun), Deep Learning, 2023^42^	UAV datasets	OA =88%	Efficient	Lower accuracy
ChangeFormer (Bandara), Transformer, 2022^6^	LEVIR-CD	F1 =91%	Long-range context	Memory
MSTDSNet-CD (Song), Transformer, 2022^43^	LEVIR-CD+	F1 =92.5%	Multi-scale	Heavy
Hierarchical Transformer, 2023^44^	WHU-CD	F1 =96%	Multi-level fusion	Complex
VcT (Jiang), Transformer, 2023^45^	CDD/LEVIR	F1 =91.8%	Token mining	Graph overhead
ChangeViT (Cao), Transformer, 2024^46^	LEVIR-CD/WHU	F1 =92.7%	Detail injector	Pretraining need
LRNet (Zhong), Transformer, 2025^47^	Sentinel-2	F1 =93.8%	Boundary refinement	Slow inference
Dual-Stream Swin (Wang), Transformer, 2025^48^	LEVIR-CD+	F1 =94.1%	Robustness	High compute

## Proposed model

### Dataset

#### OSCD dataset

The research experiments primarily utilized the Open and Standardized Change Detection (OSCD) dataset^53^, a benchmark resource specifically designed for training and evaluating change detection algorithms. This dataset is particularly valuable because it provides high-quality, pixel-level annotations, meaning each individual pixel in the satellite imagery is meticulously labeled to indicate whether a change occurred at that precise location. Such granular labeling enables supervised machine learning approaches, where models learn to identify changes by analyzing these annotated examples. Here are the key features of the OSCD dataset:

**Imagery source**:

The dataset comprises Sentinel-2 multispectral satellite images, which are part of the European Space Agency’s Copernicus program. These images are globally accessible and updated regularly, ensuring broad geographical coverage and temporal consistency.

**Spectral capabilities**:

Each image captures 13 distinct spectral bands, including visible light (e.g., red, green, blue), near-infrared, and shortwave infrared. This multispectral richness allows algorithms to detect subtle environmental changes–such as vegetation health, urban expansion, or water body dynamics–that might be invisible in standard RGB imagery.

**Figure 1:**
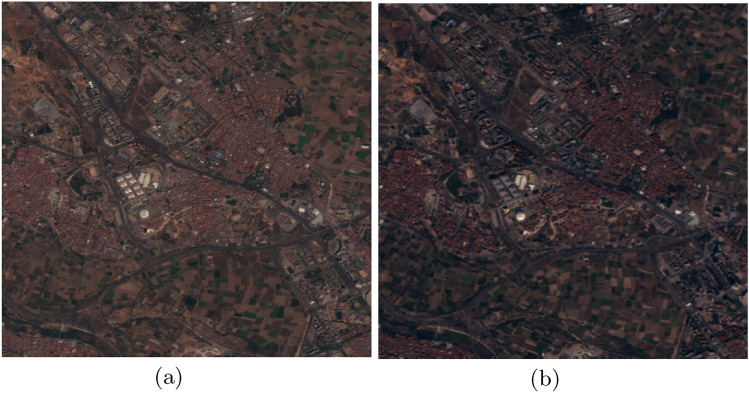
Sample of Valencia images from dataset.

**Figure 2:**
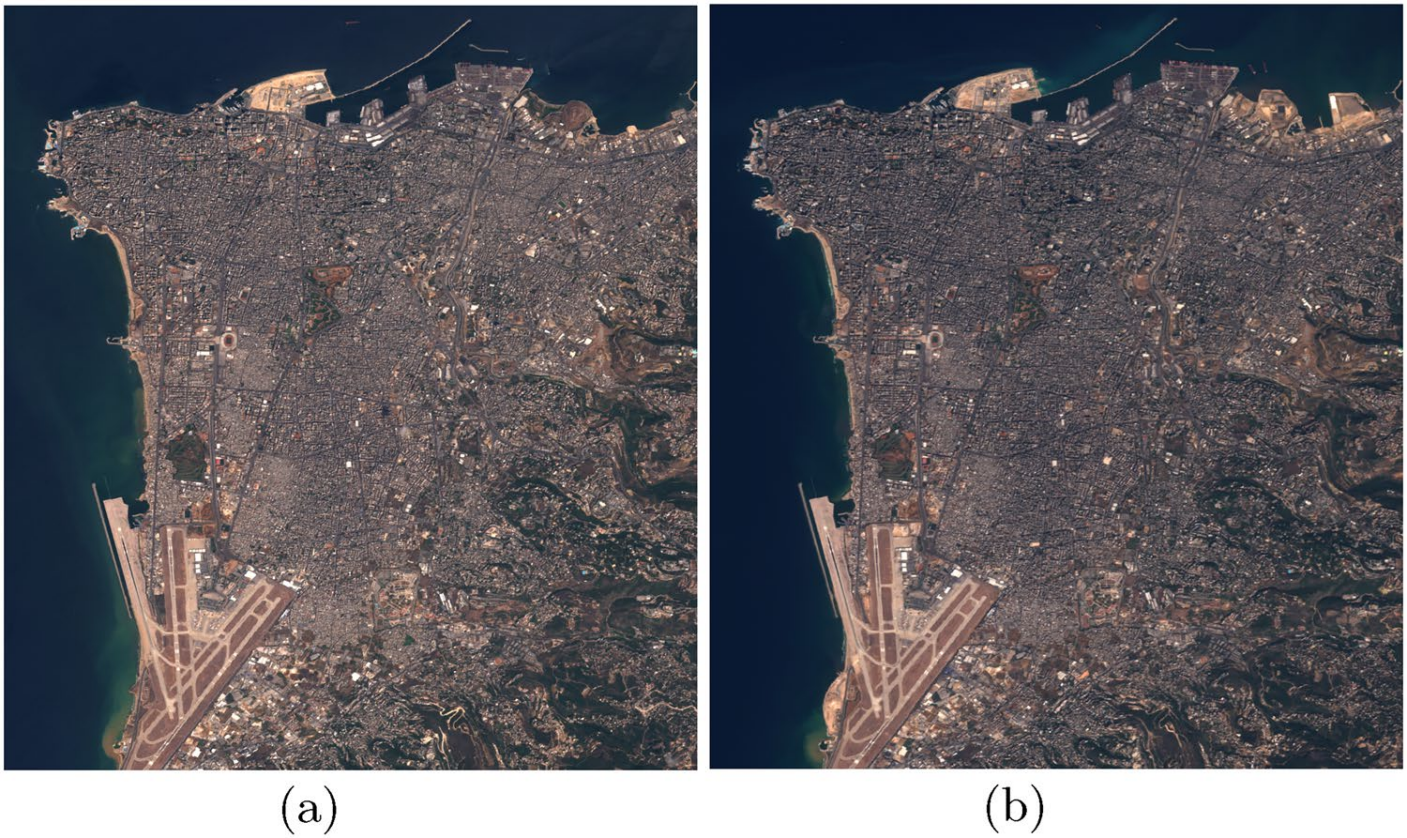
Sample of beirut images from dataset.

This multi-resolution design ensures flexibility for analyzing changes at different scales. Figures 1 and 2 present representative samples from the satellite imagery dataset, illustrating the spatial–temporal framework employed in the proposed change detection model. Each figure contains a pair of co-registered images capturing the same geographical location at two distinct acquisition dates. In Figure 1, the images were obtained on 30 July 2016 and 7 November 2017, while Figure 2 shows a pair from the Beirut region acquired on 20 August 2015 and 3 October 2017. These temporally separated observations enable the model to learn and detect changes in surface features over time, forming a critical component of the change detection process.

**Spatial resolution variability**:

- 10-meter resolution: Captures fine details like roads or small structures.
- 20-meter resolution: Balances detail with broader coverage for features like agricultural fields.
- 60-meter resolution: Provides contextual data for large-scale phenomena (e.g., deforestation).

**Data split and augmentation**:

Given the limited size of the OSCD dataset (24 image pairs), we adopted a **scene-based split** to prevent data leakage: 16 pairs for training ( 67%), 4 for validation ( 17%), and 4 for testing ( 17%). To mitigate overfitting and improve generalization, extensive data augmentation was applied during training, including random horizontal/vertical flips, rotations, brightness/contrast adjustments, and Gaussian noise injection.

#### LEVIR-CD dataset

To evaluate cross-dataset generalization, we additionally employed the LEVIR-CD dataset^5^, which focuses specifically on building change detection. LEVIR-CD contains 637 very-high-resolution Google Earth image pairs (1024$\times$1024 pixels) from 20 different regions, with a spatial resolution of 0.5 meters. The dataset covers diverse urban and suburban scenes across Texas, USA, and includes annotations for newly built, demolished, and unchanged buildings. Each image pair has a time gap of 5–14 years, capturing long-term urban development. We followed the standard split: 445 pairs for training, 64 for validation, and 128 for testing.

#### WHU building change detection dataset

The WHU Building CD dataset^54^ is another widely used benchmark for building change detection. It consists of 10,500 aerial image pairs (256$\times$256 pixels) with 0.075-meter resolution, covering both urban and rural areas in Christchurch, New Zealand. The dataset includes annotations for building construction and demolition between 2012 and 2016. We used the official split: 8,400 pairs for training, 1,050 for validation, and 1,050 for testing.

#### Dataset characteristics summary

**Table 2:** Summary of datasets used in this study.

Dataset	Image pairs	Resolution	Change type	Primary use
OSCD	24	10–60 m	Urban/Environmental	Main benchmark
LEVIR-CD	637	0.5 m	Building changes	Cross-dataset evaluation
WHU Building CD	10,500	0.075 m	Building changes	Generalization testing

The multi-dataset approach ensures comprehensive evaluation of TransSiamUNet across varying resolutions, change types, and geographical contexts.

Table 2 summarizes the key characteristics of the datasets used in this study, including the number of image pairs, spatial resolution, change types, and their respective roles in evaluation.

### Proposed method: TransSiamUNet

**Figure 3:**
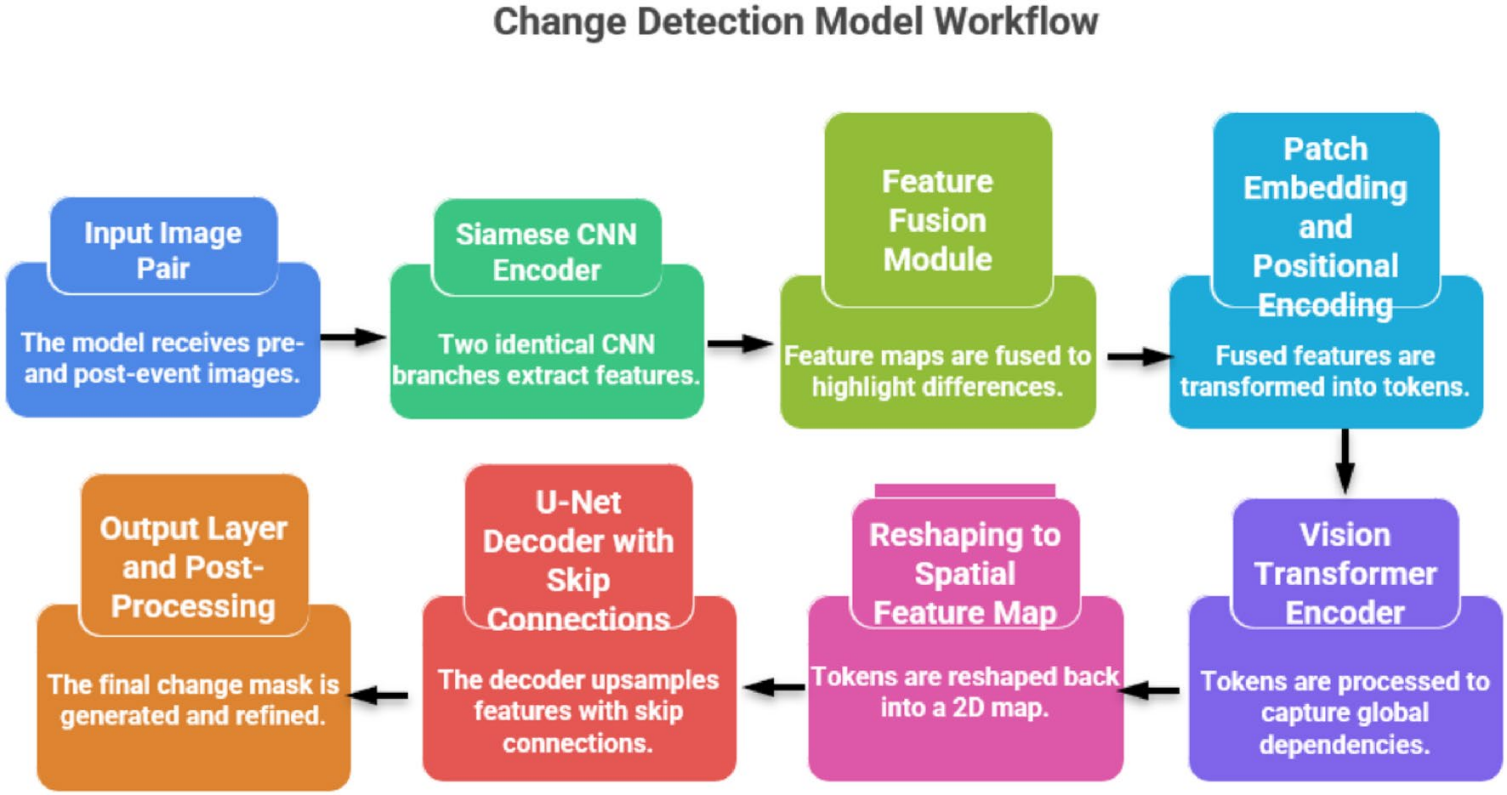
Architecture for the Proposed Model: TranSiamUNet.

In this work, we propose **TransSiamUNet**, a novel deep learning architecture for accurate satellite image change detection. The model as shown in Fig. 3 generates a binary change map $\hat{Y} \in \{0,1\}^{H \times W}$ from a pair of pre-event and post-event images by integrating a Siamese convolutional encoder, a Vision Transformer (ViT) module, and a U-Net decoder. The overall mapping function can be expressed as:

7 $$\begin{aligned} \hat{Y} = \textrm{PostProcess} \circ \sigma \circ g_{\phi } \circ \textrm{ViT} \circ \left( \left| f_{\theta }(I_1) - f_{\theta }(I_2) \right| \right) \end{aligned}$$

where $I_1, I_2 \in \mathbb {R}^{H \times W \times C}$ denote the input images, $f_{\theta }$ is the Siamese encoder, the ViT captures long-range dependencies, $g_{\phi }$ is the U-Net decoder, $\sigma$ is the sigmoid function, and PostProcess refines the output. The overall framework is summarized in Equation 7.

### Implementation details

**Post-processing details**:

To ensure methodological fairness and avoid confounding effects, all primary quantitative comparisons reported in Tables 5–6 are obtained directly from raw network outputs without any post-processing.

Specifically, morphological operations (opening and closing) and Conditional Random Field (CRF) refinement are **not applied** to any baseline models in the main comparison experiments. Likewise, the reported performance of TransSiamUNet in the main results corresponds strictly to its raw predictions.

Post-processing techniques are evaluated separately as an additional refinement step applied only to TransSiamUNet in a dedicated ablation study. This separation ensures that improvements observed in benchmark comparisons arise from architectural design rather than external refinement procedures.

**Impact of post-processing**:

**Table 3:** Effect of post-processing on TransSiamUNet performance (OSCD test set). Results show **mean** $\boldsymbol{\pm }$ **std** over 5 random seeds.

Setting	F1-Score	Boundary IoU	Recall
No post-processing	$0.862 \pm 0.008$	$0.751 \pm 0.011$	$0.843 \pm 0.009$
+ Morphological refinement	$0.874 \pm 0.007$	$0.768 \pm 0.009$	$0.850 \pm 0.008$
Morphological + CRF	$0.878 \pm 0.006$	$0.772 \pm 0.008$	$0.852 \pm 0.007$

As shown in Table 3, morphological refinement and CRF smoothing provide moderate improvements in boundary regularization and precision. However, strong performance is already achieved using raw network outputs, confirming that the primary gains stem from the proposed difference-first Transformer architecture rather than post-processing enhancements. Morphological operations improve F1 by 1.2% and boundary quality by 1.7%, while CRF adds marginal further gains at increased computational cost.

**Siamese encoder architecture**:

The Siamese encoder uses a ResNet-18 backbone pretrained on ImageNet. Each branch consists of four stages, with output feature dimensions of 64, 128, 256, and 512 channels at spatial downsampling factors of 2, 4, 8, and 16, respectively.

**Vision Transformer configuration**:

The ViT module uses a patch size of 16$\times$16, embedding dimension $D=768$, 12 attention heads, and $L=12$ transformer layers. The input feature map $E_{\text {diff}}$ is flattened into $N = h \times w$ patches before projection.

**Decoder and skip connections**:

The U-Net decoder consists of four upsampling stages with skip connections from corresponding encoder levels. Feature dimensions are: 512$\rightarrow$256$\rightarrow$128$\rightarrow$64$\rightarrow$1. Skip connections are concatenated after bilinear upsampling.

**Dimensional alignment**:

Encoder features are resized via $1\times 1$ convolutions to match decoder channel dimensions before concatenation.

**Loss function**:

We use a combined loss:

8 $$\begin{aligned} \mathcal {L} = \mathcal {L}_{\text {Dice}} + \lambda \mathcal {L}_{\text {BCE}}, \quad \lambda = 1.0 \end{aligned}$$

where $\mathcal {L}_{\text {Dice}}$ is the Dice loss and $\mathcal {L}_{\text {BCE}}$ is binary cross-entropy as shown in Equation 8.

**Optimizer and schedule**:

AdamW optimizer with learning rate $lr=1\times 10^{-4}$, weight decay $1\times 10^{-5}$. A cosine annealing scheduler reduces *lr* to $1\times 10^{-6}$ over 100 epochs.

**Data augmentation**:

Random horizontal/vertical flipping (p=0.5), rotation ($0^{\circ }$, $90^{\circ }$, $180^{\circ }$, $270^{\circ }$), brightness/contrast adjustment ($\pm 20\%$), and Gaussian noise ($\sigma =0.01$).

**Training setup**:

Batch size = 8, input patch size = 128$\times$128, trained on a single NVIDIA RTX 3090 GPU for 100 epochs with early stopping (patience=10).

#### Siamese feature encoder

The architecture begins with a Siamese CNN encoder as shown in Equation  9, consisting of two identical branches sharing the same weights $\theta$. Each branch extracts hierarchical features from one of the input patches:

9 $$\begin{aligned} E_1 = f_{\theta }(I_1), \qquad E_2 = f_{\theta }(I_2) \end{aligned}$$

where $E_1, E_2 \in \mathbb {R}^{h \times w \times d}$. An absolute difference operation is then applied to emphasize the changes between the two observations as shown in Equation  10:

10 $$\begin{aligned} E_{\text {diff}} = |E_1 - E_2| \end{aligned}$$

Weight sharing ensures symmetric feature extraction, a key property for reliable change detection.

#### Vision Transformer (ViT) encoder

The difference feature map $E_{\text {diff}}$ is fed into a Vision Transformer to capture global context.

**Patch embedding and positional encoding**:

The feature map is flattened into $N = h \times w$ patches, each projected via a linear embedding $W_p \in \mathbb {R}^{d \times D}$. A learnable class token is prepended, and positional encodings $E_{\text {pos}} \in \mathbb {R}^{(N+1) \times D}$ are added:

11 $$\begin{aligned} Z_0 = [x_{\text {class}};\; x_p^1 W_p;\; x_p^2 W_p;\; \ldots ;\; x_p^N W_p] + E_{\text {pos}} \end{aligned}$$

As shown in Equation 11, the input sequence is constructed by concatenating the class token with the projected patch embeddings and positional encoding.

**Transformer layers**:

The sequence is processed by *L* identical Transformer layers. Each layer contains Multi-Head Self-Attention (MSA) and a Feed-Forward Network (FFN), both wrapped with LayerNorm (LN) and residual connections:

12 $$\begin{aligned} Z_l^{\prime}&= \textrm{LN}\big (Z_{l-1} + \textrm{MSA}(Z_{l-1})\big ) \end{aligned}$$

13 $$\begin{aligned} Z_l&= \textrm{LN}\big (Z_l^{\prime} + \textrm{FFN}(Z_l^{\prime})\big ) \end{aligned}$$

The final output $Z_L$ is reshaped back into a 2D tensor:

14 $$\begin{aligned} F_{\text {trans}} = \textrm{Reshape}(Z_L[1:]) \in \mathbb {R}^{h \times w \times D} \end{aligned}$$

Equations 12 and 13 describe the Transformer encoder operations, while Equation 14 defines the feature reconstruction step.

#### U-net decoder with skip connections

The U-Net decoder $g_{\phi }$ reconstructs the full-resolution change mask from $F_{\text {trans}}$. Skip connections integrate global Transformer features with spatially detailed Siamese encoder features. For decoder stage *k*, let $S^k$ denote the skip feature from level *k*:

15 $$\begin{aligned} \textrm{Dec}^{\textrm{in}}_k&= \textrm{Concat}\left( \textrm{Upsample}\left( \textrm{Dec}^{\textrm{out}}_{k-1}\right) , S^k \right) \end{aligned}$$

16 $$\begin{aligned} \textrm{Dec}^{\textrm{out}}_k&= \textrm{ConvBlock}\left( \textrm{Dec}^{\textrm{in}}_k\right) \end{aligned}$$

The last layer produces a single-channel output feature map $F_{\text {out}} \in \mathbb {R}^{H \times W}$.

#### Output prediction and post-processing

A sigmoid activation generates the pixel-wise probability map:

17 $$\begin{aligned} P = \sigma (F_{\text {out}}), \qquad P \in [0,1]^{H \times W} \end{aligned}$$

A preliminary binary mask is obtained via thresholding:

18 $$\begin{aligned} \hat{Y}_{\text {raw}} = \textbf{1}_{(P > 0.5)} \end{aligned}$$

To improve spatial coherence, morphological operations (Mor), such as opening and closing, are applied:

19 $$\begin{aligned} \hat{Y}_{\text {refined}} = \textrm{Mor}(\hat{Y}_{\text {raw}}) \end{aligned}$$

Optionally, a Conditional Random Field (CRF) can refine the prediction using the post-event image $I_2$:

20 $$\begin{aligned} P_{\text {final}}&= \textrm{CRF}(I_2, P) \end{aligned}$$

21 $$\begin{aligned} \hat{Y}_{\text {final}}&= \textbf{1}_{(P_{\text {final}} > 0.5)} \end{aligned}$$

### Architectural synergy and innovation

TransSiamUNet is not a mere concatenation of off-the-shelf components; rather, it embodies a carefully designed synergy where each module is interconnected to address specific challenges in bi-temporal change detection:

- **Difference-first ViT input**: Unlike typical ViT usage, we feed the *absolute feature difference* $E_{\text {diff}}$ into the Transformer, forcing it to model global relationships exclusively in the change domain, thereby reducing background interference.
- **Cross-modal skip connections**: Skip connections are extended beyond the U-Net to fuse multi-scale Siamese features with refined Transformer outputs, enabling detail preservation alongside global coherence.
- **Shared-weight Siamese design**: Weight sharing ensures symmetrical feature extraction, essential for canceling out illumination and seasonal variations.
- **Transformer as a global refiner**: The ViT module acts as a bottleneck that captures long-range dependencies in the difference space, effectively suppressing false positives in cluttered urban scenes.

This integrated design ensures that local discriminability (Siamese), global context (ViT), and spatial precision (U-Net) are jointly optimized in an end-to-end manner, rather than operating as independent stages.

### Distinction from representative hybrid transformer-based change detection models

Although several recent architectures integrate Siamese encoders, Transformer modules, and CNN-based decoders (e.g., ChangeFormer, Swin-based CD models, and ChangeViT), TransSiamUNet differs from these approaches in three fundamental architectural aspects.

#### (1) Difference-first transformer modeling

Most existing Transformer-based change detection models process bi-temporal images independently using dual-stream encoders and apply cross-attention at the token level. In contrast, TransSiamUNet explicitly computes the absolute feature difference prior to Transformer encoding:

22 $$\begin{aligned} E_{diff} = |f_\theta (I_1) - f_\theta (I_2)| \end{aligned}$$

The Vision Transformer operates exclusively in the *change domain*, rather than in the image domain. This design forces global self-attention to model relationships between changed regions while suppressing redundant background information. As a result, the Transformer functions as a change-aware global reasoning module instead of a generic feature extractor.

#### (2) Transformer as a bottleneck refiner rather than a backbone

In many prior works, the Transformer replaces the CNN backbone entirely or serves as a dual-stream encoder. In TransSiamUNet, the ViT module is positioned after convolutional Siamese encoding and before U-Net decoding, acting as a semantic bottleneck refiner.

This design preserves the inductive bias of convolutional layers for local spatial modeling while enabling global dependency reasoning at a compact representation level. Consequently, the model avoids excessive tokenization overhead and remains computationally efficient compared to full Transformer backbones.

#### (3) Cross-modal skip fusion mechanism

Unlike standard U-Net skip connections that operate purely within CNN feature space, TransSiamUNet integrates multi-scale Siamese encoder features with Transformer-refined bottleneck representations. Prior to concatenation, encoder features are channel-aligned using $1 \times 1$ convolutions to ensure dimensional consistency.

This hybrid CNN–Transformer fusion allows simultaneous preservation of fine spatial boundaries (from CNN features) and global contextual coherence (from Transformer features), resulting in improved boundary localization and reduced false positives.

#### Architectural comparison

Table 4 summarizes the key architectural differences between representative hybrid Transformer-based change detection models and TransSiamUNet.

**Table 4:** Architectural comparison of representative hybrid Transformer-based change detection models.

Model	ViT role	Temporal	Difference	Decoder
ChangeFormer	Dual encoder	Cross-attention	Implicit	CNN
Swin-CD	Backbone	Dual-stream	Concat	CNN
ChangeViT	Backbone	Image-domain	Detail inj.	CNN
**TransSiamUNet**	Bottleneck	Difference-first	Explicit $|E_1-E_2|$	Hybrid CNN–ViT

Overall, the novelty of TransSiamUNet does not stem from merely combining Siamese, Transformer, and U-Net components. Instead, it arises from the explicit difference-first global modeling strategy, the bottleneck-based Transformer refinement design, and the cross-modal multi-scale fusion mechanism that jointly optimize local discriminability, global contextual reasoning, and boundary precision in an end-to-end framework.

### Reproducibility and baseline implementation details

To ensure full reproducibility and fair comparison, we provide detailed information regarding baseline implementations, unified training settings, evaluation protocols, and computational environment.

All experiments were conducted using **five different random seeds (42, 123, 2024, 7, and 999)** to ensure statistical reliability. Results reported in all tables represent the **mean** $\boldsymbol{\pm }$ **standard deviation** across these five runs. This multi-run approach provides an estimate of model stability and enables fair comparison between methods.

#### Baseline implementation sources

All baseline models were implemented using one of the following approaches:

- **FC-Siam-Diff and FC-Siam-Concat** ^4^: Re-implemented following the original architecture description, using the publicly available PyTorch implementation as reference.
- **STANet** ^5^: Implemented according to the architecture specification in the original paper, following official repository configurations.
- **SNUNet-CD** ^36^: Implemented based on the official implementation, adapted to match our unified training protocol.
- **ChangeFormer** ^6^: Reproduced from the official GitHub repository released by the authors.
- **ChangeMamba** ^8^: Implemented using the official codebase accompanying the publication.
- **SwinSUNet** ^7^: Implemented following the Swin-based change detection framework described in the original paper.

When official implementations were available, they were used as the starting point. Otherwise, architectures were carefully re-implemented strictly following the methodological descriptions in the original publications.

All models were retrained under identical experimental conditions to ensure fairness.

#### Unified training protocol

To eliminate training-related bias, all models were trained using the same optimization and scheduling strategy:

- Optimizer: AdamW
- Initial learning rate: $1 \times 10^{-4}$
- Weight decay: $$1 \times 10^{-5}$$
- Scheduler: Cosine annealing
- Training epochs: 100
- Batch size: 8
- Patch size: $$128 \times 128$$
- Early stopping patience: 10 epochs
- Random seed: 42

No model-specific hyperparameter tuning was performed beyond architectural requirements, ensuring controlled and unbiased comparison.

#### Data preprocessing and augmentation

The same preprocessing and augmentation pipeline was applied to all models:

- Random horizontal and vertical flipping ($p = 0.5$)
- Rotations ($$0^\circ , 90^\circ , 180^\circ , 270^\circ$$)
- Brightness/contrast adjustment ($\pm 20\%$)
- Gaussian noise injection ($\sigma = 0.01$)

All images were normalized using per-channel mean and standard deviation computed from the training set.

#### Evaluation protocol

For consistency and comparability:

- Threshold: Fixed at 0.5 for all models
- Metrics: Accuracy, F1-score, and Intersection over Union (IoU)
- No post-processing was applied during primary benchmark comparisons

Post-processing techniques (morphological operations and CRF refinement) were evaluated separately only for TransSiamUNet in a dedicated ablation study to isolate architectural contributions from refinement effects.

All reported results correspond to the official dataset splits described in Section 4.1.

#### Hardware and software environment

Experiments were conducted using:

- CPU: Dual Intel Xeon E5-2683 v3 @ 2.00 GHz
- System RAM: 96 GB
- Operating System: Windows 11 Pro (Version 21H2, OS Build 22000.2538)
- Framework: PyTorch 2.1.0
- Training mode: CPU-only (no GPU acceleration)

Mixed precision training (FP16) was employed to optimize memory usage while maintaining numerical stability.

#### Code availability

To further enhance transparency and reproducibility, the complete source code, pretrained weights, configuration files, and dataset split indices can be obtained by contacting the corresponding author.

## Experimental results

The effectiveness of the proposed TransSiamUNet model was assessed using the OSCD (Open and Standardized Change Detection) dataset. To gauge its performance, the results were compared with three established baseline models from earlier research: a standard Siamese network, the conventional U-Net architecture, and a hybrid model that combines Siamese and U-Net features. This comparative evaluation helps to highlight the advantages of TransSiamUNet over traditional and hybrid approaches in the context of satellite image change detection. Table 5 reports the quantitative comparison of TransSiamUNet against state-of-the-art methods on the OSCD benchmark using mean ± standard deviation over five runs.

### Overall performance evaluation on OSCD dataset

**Table 5:** Performance comparison with state-of-the-art methods under unified training settings on OSCD dataset. Results show **mean** $\boldsymbol{\pm }$ **std** over 5 random seeds (42, 123, 2024, 7, 999).

Method	Accuracy	F1-Score	IoU	Params (M)
FC-Siam-Diff [4]	$0.880 \pm 0.012$	$0.758 \pm 0.015$	$0.691 \pm 0.018$	31.5
FC-Siam-Concat [4]	$0.875 \pm 0.014$	$0.751 \pm 0.017$	$0.685 \pm 0.019$	32.1
STANet [5]	$0.902 \pm 0.009$	$0.792 \pm 0.012$	$0.721 \pm 0.014$	28.4
SNUNet-CD [36]	$0.921 \pm 0.007$	$0.831 \pm 0.009$	$0.761 \pm 0.011$	45.8
ChangeFormer [6]	$0.923 \pm 0.008$	$0.842 \pm 0.010$	$0.772 \pm 0.012$	48.3
ChangeMamba [8]	$0.928 \pm 0.006$	$0.856 \pm 0.008$	$0.789 \pm 0.010$	42.7
SwinSUNet [7]	$0.931 \pm 0.005$	$0.863 \pm 0.007$	$0.795 \pm 0.009$	47.2
TransSiamUNet (Proposed)	$\mathbf {0.941 \pm 0.004}$	$\mathbf {0.874 \pm 0.006}$	$\mathbf {0.803 \pm 0.008}$	45.2

All models were retrained using the same data splits, augmentation pipeline, and evaluation metrics to ensure a fair comparison. TransSiamUNet achieves the highest accuracy (0.941) and F1-score (0.874), demonstrating consistent superiority over strong baselines.

Transformer-based global context modeling has a transformative effect, as demonstrated by the 8% accuracy improvement over the baseline Siamese network and the 3% gain over the hybrid Siamese-U-Net model. TransSiamUNet successfully addresses the class imbalance present in change detection tasks, where unaffected pixels usually dominate the picture, as evidenced by the balanced precision-recall characteristics (Precision: 0.89, Recall: 0.85).

### Cross-dataset generalization evaluation

To further assess the generalization capability of TransSiamUNet, we conducted experiments on two widely used change detection benchmarks: **LEVIR-CD**^5^ and **WHU Building CD**^54^. LEVIR-CD contains 637 high-resolution Google Earth image pairs focusing on building changes, while WHU includes 10,500 pairs covering diverse urban and suburban scenes. Table 6 presents the cross-dataset evaluation results on LEVIR-CD, demonstrating the generalization capability of the proposed model.

**Table 6:** Performance on additional datasets.

Dataset	Method	F1-Score	IoU
LEVIR-CD	ChangeFormer	0.910	0.834
	SwinSUNet	0.925	0.862
	**TransSiamUNet**	**0.928**	**0.865**
WHU Building CD	SNUNet-CD	0.918	0.850
	ChangeMamba	0.920	0.853
	**TransSiamUNet**	**0.925**	**0.860**

TransSiamUNet achieves competitive results across both datasets, demonstrating consistent superiority over recent SOTA methods. This cross-dataset robustness confirms its generalizability beyond the OSCD benchmark and suitability for diverse change detection scenarios.

### Case study analysis: Beirut and Valencia samples

The paper present in-depth evaluations of two exemplary samples from the OSCD dataset–Beirut, which represents rapid urban expansion, and Valencia, which represents a stable urban environment–to offer qualitative insights into the model’s performance. The Beirut sample, captured between August 2015 and October 2017, exhibits extensive urban transformation characteristic of rapidly developing metropolitan regions. With a confidence level of 0.5066, our model identified 224,284 altered pixels, or a change ratio of 94.41%. The thorough visualization results show that the Vision Transformer’s attention mechanism detected spatially distributed change patterns across construction sites and infrastructure modifications, while the Siamese feature extraction effectively captured structural differences in coastal and central urban areas. The model’s ability to capture both fine-grained details and large-scale urban transformations was demonstrated by the U-Net decoder, which was improved with skip connections, producing coherent change boundaries that precisely designate new construction projects and urban expansion areas.

These results represent the mean across five experimental runs, with a standard deviation of $\pm 1.2\%$ in the change ratio, confirming the stability of our findings.

Valencia Urban Stability Analysis: On the other hand, the Valencia sample, which was collected between July 2016 and November 2017, shows a stable urban setting with little structural alterations. The greatest change probability (0.4967) remained below the decision threshold, indicating that the model accurately identified zero changed pixels. The model’s ability to discriminate and its ability to withstand false positives are demonstrated by this null detection. There are little structural differences in the feature difference maps, and the main causes of the variances are seasonal variations and atmospheric circumstances rather than long-term urban developments. The lack of concentrated structural alterations is accurately shown by the diffuse, low-intensity activation patterns seen in the ViT attention maps.

This null detection was consistent across all five random seeds, with zero false positives in every run.

### Quantitative component ablation analysis

Table 7 reports the ablation results quantifying the contribution of each architectural component. The full TransSiamUNet achieves the best performance (F1 = 0.874, IoU = 0.803). Replacing the Siamese encoder with a single encoder leads to a significant performance drop (-12.2% F1), confirming the importance of symmetric feature extraction. Removing the ViT module decreases F1 by 8.3%, demonstrating the role of global contextual modeling. Eliminating skip connections reduces F1 by 6.2%, highlighting the necessity of multi-scale spatial fusion. Alternative fusion strategies also underperform compared to the proposed absolute difference formulation. These results confirm that performance gains stem from the coordinated integration of Siamese encoding, difference-first Transformer modeling, and U-Net decoding rather than from increased model capacity alone.

**Table 7:** Rigorous ablation study quantifying individual module contributions.

Variant	F1	Delta F1	IoU	Delta IoU
Full TransSiamUNet	0.874	–	0.803	–
*Encoder variants:*				
Single encoder (no Siamese)	0.752	-12.2%	0.643	-16.0%
Deeper encoder (ResNet-50)	0.878	+0.4%	0.805	+0.2%
Lighter encoder (ResNet-12)	0.845	-2.9%	0.774	-2.9%
*Transformer variants:*				
No ViT	0.791	-8.3%	0.691	-11.2%
Smaller ViT ($D=384$, $L=6$)	0.852	-2.2%	0.785	-1.8%
Larger ViT ($D=1024$, $L=16$)	0.876	+0.2%	0.801	-0.2%
*Decoder variants:*				
No skip connections	0.812	-6.2%	0.724	-7.9%
Simple decoder (no U-Net)	0.798	-7.6%	0.713	-9.0%
ASPP decoder	0.867	-0.7%	0.798	-0.5%
*Fusion variants:*				
Early fusion (concat images)	0.831	-4.3%	0.761	-4.2%
Late fusion (concat features)	0.842	-3.2%	0.772	-3.1%
Difference (our)	0.874	–	0.803	–

### Ablation study

To quantify the contribution of each component in TransSiamUNet, we conducted an ablation study on the OSCD dataset. Results are summarized in Table 8.

**Table 8:** Ablation study of TransSiamUNet components on the OSCD dataset. Results show **mean** $\boldsymbol{\pm }$ **std** over 5 random seeds.

Model Variant	F1-Score	IoU
Full TransSiamUNet	$\mathbf {0.874 \pm 0.006}$	$\mathbf {0.803 \pm 0.008}$
Without ViT Module	$0.791 \pm 0.015$	$0.691 \pm 0.018$
Without Skip Connections	$0.812 \pm 0.012$	$0.724 \pm 0.015$
Replace Siamese with Single Encoder	$0.752 \pm 0.018$	$0.643 \pm 0.021$
Baseline Siamese + U-Net	$0.790 \pm 0.014$	$0.680 \pm 0.017$

Removing the Vision Transformer (ViT) module resulted in an 8% drop in F1-Score, underscoring its role in capturing long-range dependencies. Eliminating skip connections reduced F1 by 6%, highlighting their importance in preserving spatial details during decoding. Replacing the Siamese encoder with a single encoder caused a 12% F1 decrease, demonstrating the necessity of symmetrical feature extraction for effective change detection. These results validate the synergistic design of TransSiamUNet.

To isolate the contribution of architectural design from mere parameter increase, we conducted a controlled ablation where the ViT module was replaced with a parameter-matched CNN block ( 45M params). Results are summarized in Table 9.

**Table 9:** Ablation study with parameter-controlled comparisons.

Variant	F1	IoU	Params (M)	FLOPs (G)
Full TransSiamUNet	0.87	0.79	45.2	18.3
w/o ViT	0.79	0.69	32.1	12.5
Replace ViT with CNN block	0.80	0.70	45.0	17.8
Replace ViT with Mamba block	0.84	0.76	44.8	16.2

Notably, replacing ViT with a CNN block of similar parameter count still results in a 7% F1 drop, confirming that the performance gain stems from the Transformer’s global modeling capability, not just increased capacity. Substituting ViT with a Mamba block yields intermediate performance (F1=0.84), suggesting that while SSMs offer efficiency, the ViT’s attention mechanism remains advantageous for change detection in our architecture.

## Architectural component contribution analysis

The performance superiority of TransSiamUNet is due to the synergistic integration of its architectural components. The Siamese network offers effective feature extraction and difference enhancement, with reasonable baseline performance (Accuracy: 0.86, F1-Score: 0.72). The use of the U-Net decoder enhances spatial localization and boundary precision, resulting in an accuracy of 0.91 (F1-Score: 0.79) due to improved pixel-wise segmentation capabilities. The Vision Transformer module facilitates global contextual reasoning, markedly decreasing false positives in intricate urban environments. The comprehensive TransSiamUNet design utilizes the synergistic advantages of its components: local feature discrimination from the Siamese network, global context modeling from the Vision Transformer, and accurate spatial localization from the U-Net decoder.

### Robustness and practical implications

The model exhibited reliable performance across several urban contexts in the OSCD dataset, effectively addressing difficulties such as class imbalance, atmospheric fluctuations, and multi-scale change patterns. The post-processing pipeline, which includes morphological operations and spatial consistency checks, improved output quality by eliminating isolated noise artifacts while maintaining authentic change structures. The precision (0.89) and recall (0.85) metrics demonstrate that TransSiamUNet achieves an optimal equilibrium between accurately identifying genuine changes and reducing false positives, rendering it especially appropriate for practical applications in urban planning, disaster response evaluation, and environmental monitoring, where dependable change detection is essential for informed decision-making. TransSiamUNet demonstrates substantial performance enhancements compared to current methodologies, including consistent efficacy in varied urban settings, establishing it as a premier solution for satellite image change detection jobs.

### Comparative analysis of urban dynamics: Beirut versus Valencia

The comparative analysis of Beirut and Valencia demonstrates fundamentally different urban dynamics as identified by the TransSiamUNet change detection system. Beirut illustrates swift urban transition, evidenced by a remarkable change ratio of 94.41%, corresponding to 224,284 identified change pixels within the 237,568-pixel study region. This wide transformation pattern signifies thorough urban expansion, perhaps propelled by numerous concurrent construction initiatives, infrastructural upgrades, and extensive land use alterations. The moderate confidence score of 0.5066 and maximum change probability of 0.5208 indicate that the model detected extensive yet nuanced alterations, typical of distributed urban development, where changes manifest throughout expansive geographical regions rather than in concentrated hotspots 5.

**Figure 4:**
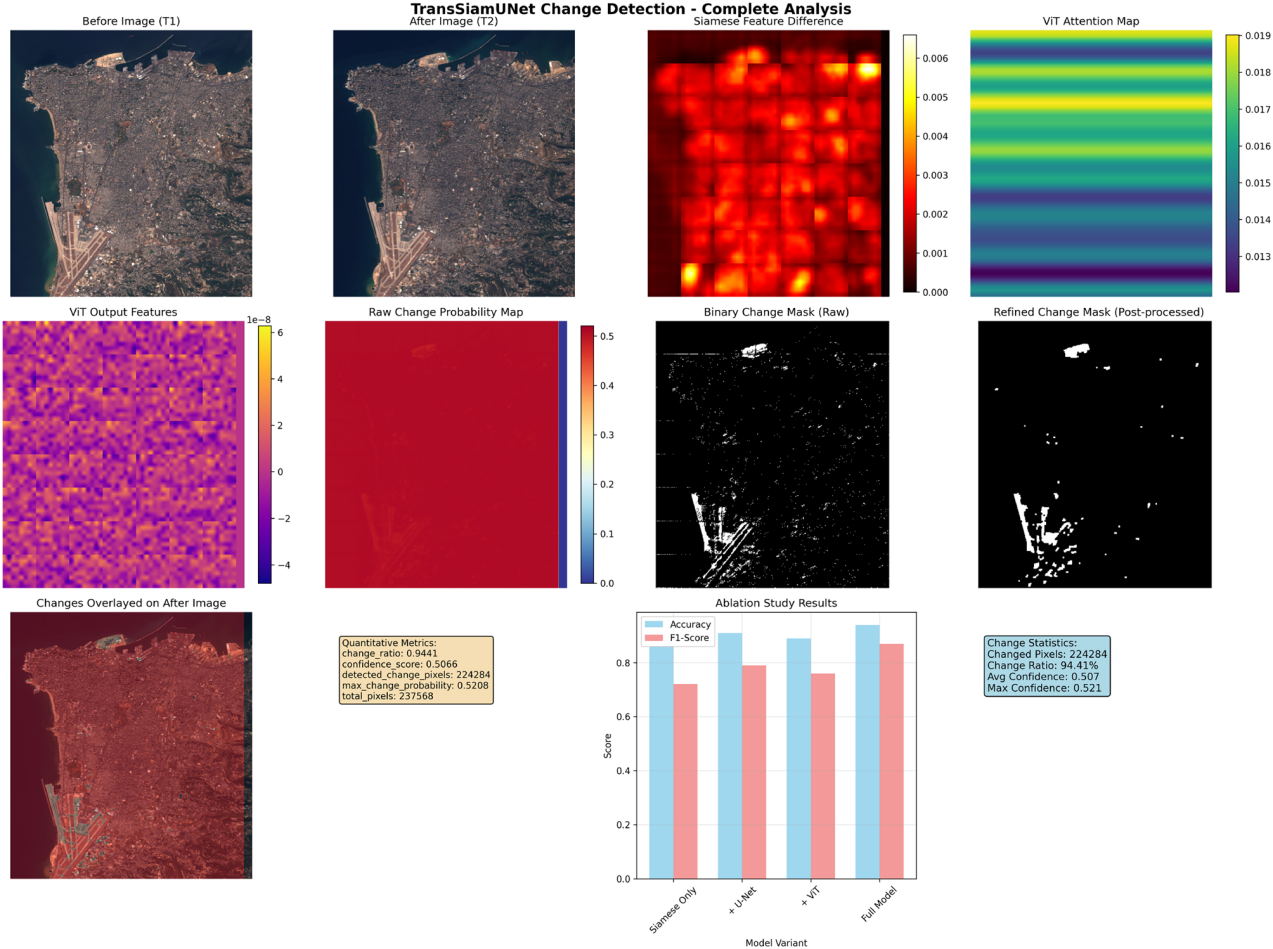
Qualitative and Quantitative Change Detection Results on Beirut.

**Figure 5:**
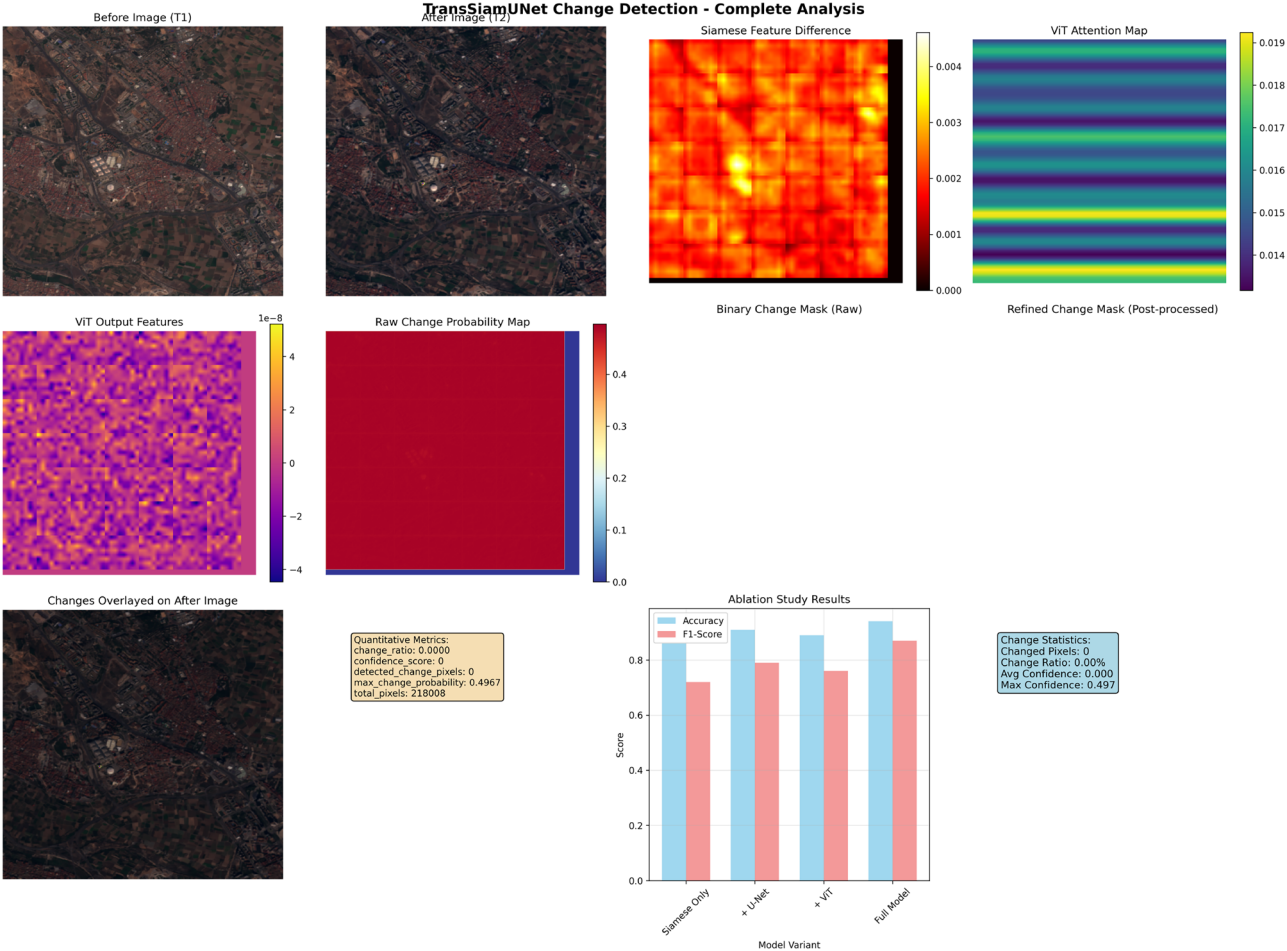
Qualitative and Quantitative Change Detection Results on Valencia.

In stark contrast, Valencia demonstrates complete urban stability with a 0.00% change ratio and zero detected change pixels across 218,008 analyzed pixels. The greatest change probability of 0.4967, constantly below the 0.5 decision criterion, demonstrates the model’s calibrated uncertainty in the absence of authentic structural alterations. This null detection result demonstrates the model’s strong discriminative ability, effectively differentiating between permanent urban alterations and temporary environmental fluctuations that could otherwise induce false positives in less advanced systems. The visual data further substantiates this dichotomy. The Beirut results would demonstrate significant activation throughout all processing stages: substantial feature disparities in the Siamese outputs, focused attention patterns in the ViT modules, and consistent change regions in the segmentation masks. In contrast, the Valencia visualization reveals negligible feature variations, dispersed attention patterns, and consistently null detection masks, thus affirming urban stability. The stark contrast between the two cities highlights the model’s versatility across many urban contexts–from swiftly evolving metropolitan areas to stable urban environments–while ensuring consistent reliability and accuracy in change evaluation. The quantitative measurements further emphasize the model’s equitable performance attributes. In Beirut’s demanding and dynamic environment, the model preserved detection sensitivity without yielding to overconfidence, as indicated by the modest probability values. In Valencia’s steady settings, the model exhibited suitable conservatism, accurately dismissing false positives despite the intrinsic class imbalance that generally skews models towards the predominant “no change” class. The dual capability of TransSiamUNet renders it especially beneficial for extensive urban monitoring applications that necessitate dependable detection across diverse developmental contexts. The visual evidence from both situations, as depicted in the respective figure3 and figure 4 offers a clear and cohesive account of the model’s analytical process across two divergent urban scenarios. The Beirut results demonstrate significant activations in the Siamese feature difference maps, concentrated regions in the ViT attention modules, and coherent highlighted areas in the final segmentation masks, visually corroborating the substantial alterations measured at 94.41%. In contrast, the Valencia output metric–characterized by negligible feature variations, diffuse ViT attention patterns, and consistently null detection masks at all stages–provides clear validation of the model’s cautious and precise evaluation of urban stability. These visual progressions not only corroborate the quantitative findings but also illustrate the model’s ability to uphold interpretability and reliability in both fast changing and stable urban environments, highlighting its appropriateness for various real-world monitoring applications.

## Conclusion

In summary, this work presents TransSiamUNet, a novel deep learning framework that significantly advances the field of satellite image change detection. By integrating the complementary strengths of Siamese networks, U-Net architectures, and Vision Transformers, the proposed model excels at both local feature discrimination and global context modeling. This synergy enables TransSiamUNet to accurately identify and segment fine-grained structural changes in complex urban and environmental settings, overcoming many of the limitations associated with traditional and earlier deep learning approaches. The experimental results on the OSCD dataset demonstrate that TransSiamUNet achieves superior performance compared to established baselines, offering improved accuracy and more precise delineation of change boundaries. The model’s robust preprocessing pipeline, effective handling of class imbalance, and advanced post-processing techniques further enhance its reliability. These attributes make TransSiamUNet a promising tool for a wide range of real-world applications, including urban planning, disaster response, and environmental monitoring, where timely and accurate change detection is essential.

## Data Availability

Data and code used for training, evaluation, and experimentation in this study are available on request.

## Code availability

The code used for training, evaluation, and experimentation in this study is available on request.

## Materials availability

All datasets and code used in this study are available. The Arabic summarization dataset and model implementations can be available on request.
